# Enhanced Biosorption of Nickel Ions on Immobilized Surface-Engineered Yeast Using Nickel-Binding Peptides

**DOI:** 10.3389/fmicb.2019.01254

**Published:** 2019-06-26

**Authors:** Hua Li, Wei Dong, Yong Liu, Haiyan Zhang, Gang Wang

**Affiliations:** ^1^Institute of Microbial Engineering, Henan University, Kaifeng, China; ^2^School of Life Sciencel, Henan University, Kaifeng, China; ^3^College of Chemistry and Chemical Engineering, Henan University, Kaifeng, China

**Keywords:** nickel-binding peptide, biosorption, phage peptide library, surface-engineered yeast, immobilization

## Abstract

Three nickel-binding peptides were screened from a phage peptide library and displayed separately or in combination with surface-engineered *Saccharomyces cerevisiae* EBY100. The sorption of nickel ions on the surface of yeast cells increased with the increasing number of nickel Ni(II)-binding peptides displayed. The combined expression of the three peptides by EBY100/pYD1-N123 demonstrated the highest sorption of Ni(II) (2.603 ± 0.004 g g^−1^, dry weight) and an enhanced sorption capacity of 60.15%, compared to *S. cerevisiae* EBY100. An orthogonal test for yeast immobilization was designed. A maximum sorption capability of 68.62% was observed for a treatment at 25°C with 2.0% calcium chloride and 3.0% sodium alginate.

## Introduction

Diverse adverse results of heavy metal including Ni exposure have been reported in humans and animals (Zhang et al., 2014; Guo et al., 2015; Biscéré et al., 2017). The conventional treatment removal of Ni has its own limitations; biosorption by microorganisms is recognized as a potential alternative to replace chemical technology (Soares and Soares, 2012; Kamika and Momba, 2013). In particular, the combination of cell-surface display and microbial adsorbents has become a powerful strategy for environmental management. The expression of heterologous metal-binding proteins or peptides on the cell surface through microbial cell-surface display systems enables specific binding of heavy metals and improves the resistance of microorganisms to metal and sorption of cells. In recent years, the combination of microbial metal-binding protein with surface-expressed system has been utilized to treat heavy metal wastewater. For example, a Hg^2+^-binding protein that was surface-expressed on the ice-nucleation protein of *Escherichia coli* binds six-fold more than the wild-type strain (Bae et al., 2003). There are several types of microbial surface display systems (Lee et al., 2003; Desvaux et al., 2006; Cindy et al., 2008). Compared to bacterial systems, yeast display system has the merit of the protein modification and processing in yeast occurs after translation, and the heterologously expressed protein cannot be easily degraded by proteases (Kuroda and Ueda, 2013; Li et al., 2017).

In addition to considering how to improve the sorption of heavy metal ions, another problem is how to develop a specific bioadsorbent that efficiently adsorbs the target metal from complex wastewater. The problem can be solved with the phage peptide library screening technology (Cindy et al., 2008; Bratkovicˇ, 2010). This approach allows for rapid and combinatorial screening of sequences that can display high affinities to targets and bind to any specific target, including inorganic materials (Yang et al., 2018), proteins (Zarei et al., 2017), membrane receptor modulators (Molek et al., 2011), cells (He et al., 2017), viruses (Castel et al., 2011), and nanoparticles (Li et al., 2011).

However, the application of recombinant bioadsorbents can be problematic due to the complexity of wastewaters. To solve this problem, one strategy is cell immobilization (Juarez et al., 2012). Because the sorption of immobilized cells depends on the peptide’s metal chelation, which does not rely on cell activities, even if the preparation of cells lost vitality, surface display peptides still can absorb the metal ion. This not only solves the toxicity of complex pollutants to microbial cells but also allows for continuous operation in industrial applications.

In the study, three Ni-binding peptides screened from a phage random peptide library were transformed on the surface of *Saccharomyces cerevisiae* cells separately or commonly *via* gene recombination; then, the bioadsorbents were treated by cell immobilization.

The aim of this study is to demonstrate whether the recombinant *S. cerevisiae* cells with three Ni-binding peptides transformed onto the surface which were immobilized in the alginate gel can perform as a high-capacity bioadsorbents for nickel adsorption from wastewater.

## Materials and Methods

### Media and Solutions

LB medium contained (l^−1^) 10 g Bacto-tryptone, 5 g yeast extract, and 5 g NaCl. The IPTG/X-gal stock, contained 1.25 g isopropyl-β-D-thiogalactoside (IPTG) and 1 g X-gal (5-bromo-4-chloro-3-indolyl-β-D-galactoside), which were mixed together in 25 ml DMF (dimethyl formamide) and then stored at −20°C. Top agar contained (l^−1^) 10 g bacto-tryptone, 5 g yeast extract, 5 g NaCl, and 7 g agar, which was dispensed in 50 ml aliquots. To make LB^+Tet^ plates, LB medium and 15 g l^−1^ agar was autoclaved and cooled to <70°C, after which 1 ml tetracycline stock was added. Plates were stored at 4°C in the dark. YNB medium contained (l^−1^) 20 g glucose and 6.7 g yeast nitrogen base with ammonium sulfate without amino acids. YNB-CAA medium (l^−1^) contained 5 g casamino acids and 6.7 g yeast nitrogen base with ammonium sulfate. Blocking buffer contained 0.1 M NaHCO_3_ (pH 8.6) and 5 g l^−1^ BSA and was stored at 4°C. TBS contained 50 mM Tris-HCl (pH 7.5) and 150 mM NaCl, and 0.1% TBST was made by the addition of 0.1% Tween 20.

DNA Leader Marker was purchased from TransGen Biotech (Beijing). Most chemicals and reagents were of molecular biology or HPLC grade. Ultrapure water was used throughout the study and was obtained using a Millipore water purification system (Element A10, Chengdu youyue, China).

### Preparation of Ni-NTA Agarose

Ni-NTA Agarose (R90101, Invitrogen, USA) was used as binding substrate and eluted with TBS, which can washed off Ni iron served as a control containing 0.5 M EDTA (pH 8.0).

### Phage Display Library Screening

#### Phage Display Biospanning

The Ph.D.-12 peptide library kit (E8110S, New England Bio Labs, USA) was used for panning experiments. A solution of 100 μg ml^−1^ Ni-NTA agarose was prepared in 0.1% TBST, while the control was made in 0.1 M NaHCO_3_ (pH 8.6), each of which was mixed with 10 μl of the phage library suspension and incubated for 1 h at 25°C. Unbound or loosely bound phages were removed by 10 washes with TBS. The tightly bound phages were eluted with 1 ml of 20 mmol imidazole followed by a subsequent elution with 200 mmol imidazole. Next, the eluted phages were amplified using *E. coli* 2738 and purified by a polyethylene glycol precipitation. This procedure was repeated four times using increasing amounts of the TBST buffer (containing 0.3, 0.4, and 0.5% Tween 20 sequentially from second to the fourth round). Four rounds of screening were performed to obtain nickel-binding peptides with high specificity and affinity. The eluted phages were diluted and titrated on *E. coli* 2738 plates. More than 20 plaques were selected and analyzed by DNA sequencing. A detailed procedure can be found in the Ph.D.™ Phage Display Library kit instruction manual.

#### Phage Tittering

*E. coli* strain ER2738 was used to amplify the phages. When the cultures reached mid-log phase, 200 μl of culture was dispensed into pre-warmed microcentrifuge tubes, 10 μl phage dilutions was added and the mixture was briefly vortexed, then incubated at room temperature for 3 min. The infected cells were transferred to culture tubes containing 45°C top agar, vortexed briefly, then immediately poured onto a pre-warmed LB/IPTG/X-gal plate and incubated overnight at 37°C. The number of plaques was determined with plaque forming units (pfu L^−1^).

Phages were amplified in *E. coli* ER2738 cultured in LB that was supplemented with tetracycline. After shaking at 37°C for 4.5 h, bacteria were pelleted by centrifugation (30 min, 1,811 g). The phages in the supernatant were precipitated overnight at 4°C with 25% (w v^−1^) polyethylene glycol 6,000 in 2.5 mol L^−1^ NaCl (PEG-NaCl). Next, the phages were pelleted by centrifugation and resuspended in 1 ml PBS. Residual bacteria were pelleted by centrifugation (5 min, 15,700 g), and the phage precipitation with PEG-NaCl was repeated for 3 h at 4°C.

#### Amplification and Purification of Phages

Phages were amplified in *E. coli* ER2738 in LB, supplemented with tetracycline (20 g L^−1^). After 4.5 h of shaking at 37°C, bacteria were pelleted by centrifugation at 30 min, 1,811 g. The phages in the supernatant were precipitated overnight at 4°C in 2.5 mol L^−1^ PEG-6000-NaCl. The P/N value was calculated using the following equation: P/N = the phage titre in the sample hole (P)/the phage titre in the control hole (N).

#### Enzyme-Linked Immunosorbent Assay

An ELISA assay was conducted for target binding. The selected phage was diluted with 0.1% TBST, mixed with Ni-NTA agarose (sample) and NTA agarose without Ni ion (CK), incubated gently at room temperature for 60 min, and washed two times with 0.1%TBST and four times with 0.05% TBST. HRP-IgG diluted with PBS at 5,000^−1^ proportion was added into samples and incubated gently at room temperature for 60 min, then washed six times with 0.05% PBST. About 200 μl color solution was added for the color rendering for 10 min; finally, 1 mol^−l^ H_2_SO_4_ was added to terminate the chromogenic reaction. The ratio of OD_450nm_ (sample) to OD_450nm_ (CK) was measured to determine the target specificity (Lei et al., 2016).

### Expression in *S. cerevisiae* EBY100

#### Construction of Plasmids and Transformation into *S. cerevisiae* EBY100

*S. cerevisiae* EBY100 and the display vector pYD1 were purchased from invotrogen. Three Ni-binding peptide-encoding genes were synthesized artificially separately or commonly (in sequence of N1-N2-N3) with *Eco*R I and *Xho* I restriction enzyme sites. The PCR product was digested with the appropriate restriction enzymes and ligated to the display vector pYD1. The recombinant plasmids were named pYD1-N1, pYD1-N2, pYD1-N3, and pYD1-N123.

*S. cerevisiae* EBY100 was cultured in YNB medium with amino acids (100 mg l^−1^ leucine and 100 mg l^−1^ tryptophan) at 30°C. When the optical density at 600 nm (OD600) reached 0.6, the cells were harvested to prepare competent cells for transformation using the heat-shock method. Ten microliters of recombinant plasmid DNA, 700 μl 1 × LiAc/10% PEG-3350/1 × TE, 10 μl salmon extract DNA, and EBY100 competent cells were mixed and incubated at 30°C for 30 min in a water bath. Next, 88 μl of DMSO was added, mixed, and the mixtures were incubated at 42°C for 7 min. After heat-shock transformation, the cells were grown on YNB (Trp^−^ and Leu^+^) medium at 30°C for 72 h to select for the positive clones. Further confirmation was performed using colony PCR amplification and a dual digestion with plasmid DNA isolated from the clones that grew on YNB (Trp^−^ and Leu^+^) plates. The recombinant plasmid DNA was retransformed into *E. coli* 116 to verify the correct expression. The transformants containing the correct plasmid DNA were grown in YNB-CAA medium containing 20 g L^−1^ glucose at 30°C overnight with shaking. Next, the cells were collected and grown at 25°C with shaking in YNB-CAA medium containing 20 g L^−1^ galactose to an OD600 of 1.0. The cells that displayed the Ni-binding peptide were collected, and the binding capacity of adsorbed Ni(II) was calculated as the weight of absorbed Ni(II) (mg)/dry weight of yeast sample (g).

#### Western Blot Analysis

A western blot assay was carried out to test the Ni-binding peptides display on the yeast surface. Freshly harvested yeast cells were grown at 30°C with shaking in YNB-CAA medium containing 20 g L^−1^ galactose to an OD600 of 1.0, and the cells that displayed the Ni-binding peptide were collected. Proteins were separated by TRICINE-SDS-PAGE, then were transferred to a 0.2 μm PVDF membrane (Millipore, USA), and detected with 1:10 diluted antibody (Zhang et al., 2018a,b).

### Immobilization of Absorbent and Selectivity to Other Heavy-Metal Ions

First, recombinant cells and wild-type *S. cerevisiae* EBY100 were cultivated in YNB medium at 30°C, 200 rpm. When the optical density of the cultures at 600 nm reached 0.6, the cells were harvested to prepare immobilized cells. Cells were mixed with a series of sodium alginate (Sigma) solutions, from 0.5 to 4% (w v^−1^), to yield cell-embedded beads. The sodium alginate beads were prepared by dripping the well-mixed sodium alginate and cell mixture from a needle into pre-prepared 0.1 M CaCl_2_ and fixed for 24 h. Finally, the sodium alginate beads containing cells were washed with phosphate buffer and sterile water and stored at 4°C. The concentration of sodium alginate, calcium chloride, and temperature were varied to determine the optimal immobilization conditions.

About 7 g l^−1^ NiSO_4_ was added into the medium to elevated the Ni(II) sorption capability. The Ni(II) sorption capability was calculated according to a standard curve using an inductive coupled plasma emission spectrometer. Based on the single factor data, an orthogonal test with three factors and three levels was designed to optimize the immobilization parameters (Zhang et al., 2017). The binding capability of Ni(II) = the weight of absorbed Ni(II) (g)/total weight of Ni(II) (g) in the medium. The viability of absorbents was tested by the colony counting method. 10 g L^−1^ NiSO_4_ was used in disc diffusion assay to elevate certain resistance of yeast strains. The effect of pH as a variable was examined in a single factor test that showed the most sorption capacity at pH 7. As a result, the pH was set to 7 in the orthogonal test.

For selectivity test, solutions were replaced by other metal ions including Cr(III), As(III), Pb(II), and Cd(II). The binding capability = the weight of absorbed metal ions (g)/total weight of metal ions (g) in the medium.

### Statistical Analysis

All the experiments were performed in triplicate, and all data throughout this study are reported as the means with standard deviation (SD) with *p* < 0.05 considered as statistically significant.

## Results and Discussion

### Screening of Ni-binding Peptides From the Ph.D.-12 Peptide Library

Phage display is a versatile technology for the discovery of peptides which bind to any desired target (Cindy et al., 2008; Bratkovicˇ, 2010). These targets consist of a great diversity of inorganic and organic materials, including nanomaterials, carbohydrates, cells, organs, and antigens (Cui et al., 2010; Chung et al., 2011). In our study, the Ph.D.-12 Peptide Library was used to identify Ni-binding peptides. Four rounds of consecutive biopanning were carried out using Ni(II)-NTA agarose as a binding target, with cell surface-binding phages recovered by cell lysis in each round. The P/N value was used to reflect the ability of specific clones for each round to bind Ni(II); when the P/N value is above 50 or higher, the chance of obtaining a specific sequence is increased. The observed P/N value after the first, third, and fourth biopanning round was 1.4, 58, and 956, respectively. Each biopanning and amplification is a specific phage peptide enrichment process. After the fourth biopanning, 20 plaques from the third round of biopanning were randomly selected, PCR amplified, and sequenced. Using an ELISA method, the target specificity of the sequenced peptides from the phage clones for Ni(II) was evaluated. The results showed that the ratio of OD450nm was above 4, which means the selected peptides can binding to Ni(II) specially.

**Figure 1:**
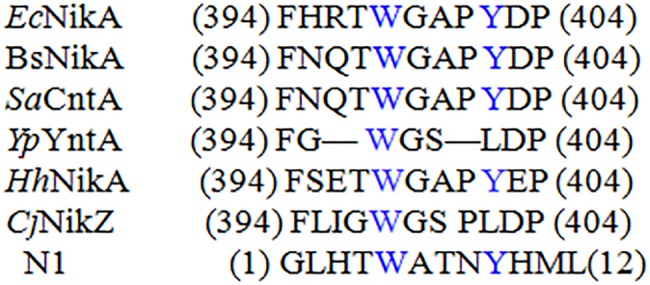
Sequence alignments of described Ni-BPs with N1. Each sequence was independently aligned with EcNikA sequence. Residues conserved with EcNikA binding site are depicted in blue.

As shown in Table 1, a Ni(II)-binding peptide with a sequence of GLHTWATNLYHM (named N1) was obtained through the repeated positive screening against Ni(II) and occurred at a much higher frequency than the other two Ni-binding peptides obtained in the biopanning process. N1 contains a Ni(II)-binding motif that is identical to the partial of the Ni(II)-binding proteins (Ni-BPs). Ni-BPs are in a subclass of Ni(II) ATP-binding cassette (ABC)-type transporters, which are responsible for the acquisition and binding of Ni(II) and its import through the inner membrane. N1 was aligned with partial Ni-binding motifs of Ni-BPs from different microorganisms, including *Ec*NikA from *E. coli* (Cherrier et al., 2008), *Hh*NikA from *Helicobacter hepaticus* (Benoit et al., 2013), *Bs*NikA from *Brucella suis* (Jubier-Maurin et al., 2001), *Sa*CntA from *Staphylococcus aureus* (Remy et al., 2013), NikZ from *Campylobacter jejuni* and *Yp*YntA from *Yersiniae* (Sebbane et al., 2002), all of which possess a Ni-binding motif. In the Ni-binding site, TRP398 is involved in CH-p and p-stacking interactions, while Tyr402 stabilizes structural water (Figure 1; Cherrier et al., 2012). N2 (HAVSPTLPAYSK) had a similarity with a Ni-binding motif, ^94^H^109^ AVSEGTKAVTKYTSSK^125^, from histone H2B. The motif was investigated in end-blocked peptides for the Ni-binding ability (Nunes et al., 2010). On the basis of NMR measurements, a well-resolved solution structure for the binding site of the H2B_94–125_-Ni(II) complex was determined. It has been shown that Ni binding affects the C-terminal tail of the peptide, forcing it to approach the coordination plane.

**Table 1:** Screened nickel sorption peptides from peptides library.

No.	Amino acid sequence	Frequency	KD	The ratio of D_450_
N1	GLHTWATNLYHM	6	1.44	5.45 ± 0.55
N2	HAVSPTLPAYSK	3	1.27	5.04 ± 0.71
N3	SGVYKVAYDASR	3	1.32	4.73 ± 0.24

Frequency means number of times independently isolated from plaques.

### Display of Ni-binding Peptides on *S. cerevisiae* EBY100 and Ni(II) Sorption

The yeast display system uses the a-agglutinin receptor of *S. cerevisiae* to display foreign proteins on the cell surface. The a-agglutinin receptor consists of two subunits encoded by the AGA1 and AGA2 genes. The Aga1 protein (Aga1p, 725 amino acids) is secreted from the cell and becomes covalently attached to β-glucan in the extracellular matrix of the yeast cell wall. The Aga2 protein (Aga2p, 69 amino acids) binds to Aga1p through two disulfide bonds and remains attached to the cell through its contact with Aga1p after secretion. The N-terminal portion of Aga2p is required for attachment to Aga1p, while proteins and peptides can be fused to the C-terminus for presentation on the yeast cell surface. Vector pYD1 is 5.0 kb which designed for expression and display of proteins on the extracellular surface of *S. cerevisiae*. The vector contains the AGA2 gene from *S. cerevisiae* which encodes one of the subunits of the a-agglutinin receptor. Fusion of the gene of interest to AGA2 allows secretion and display of the protein on the cell surface.

In our study, plasmid pYD1 contains genes encoding the peptides N1, N2, and N3, or all the three in sequence of N1-N2-N3 (N123) were transformed into *S. cerevisiae* EBY100 (EBY100/pYD1-N1, EBY100/pYD1-N2, EBY100/pYD1-N3, EBY100/pYD1-N123) (Kuroda and Ueda, 2013). The plasmid pYD1 was also transformed to make the EBY100/pYD1 (WT) strain used as a control.

**Figure 2:**
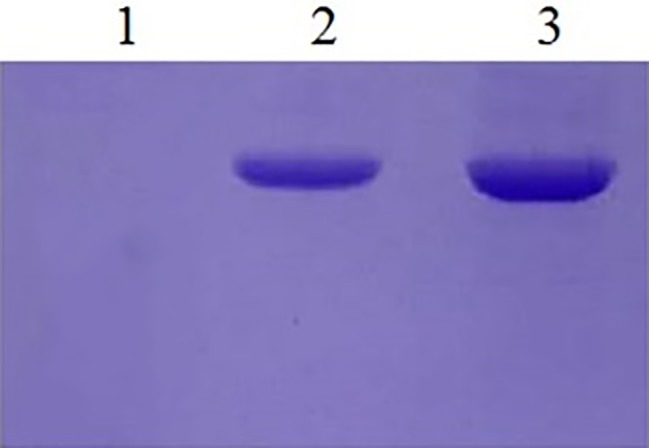
Western blotting of yeast recombinants. Lane 1: The yeast EBY100/pYD1 without insert fragment. Lane 2: Recombinants of EBY100/pYD1-N1 with N123 Ni(II)-binding peptides. Lane 3: Recombinants of yeast EBY100/pYD1-N1 with all three Ni(II)-binding peptides.

EBY100 cannot be grown in cultures lacking tryptophan, while recombinant EBY100 contains pYD1 plasmids that can be grown in cultures lacking tryptophan and the transformants were screened on the cultures lacking tryptophan. However, it is not certain that if recombinant yeast cells really produce Aga2p-N1, -N2, -N3, and -N123. Then, the expression of Ni-binding peptides was detected by a colony PCR assay. Because the Ni-binding peptides are too short to detect, gene fragments were sequenced after the colony PCR assay, the results showed that the N1, N2, N3, and N123 inserted correctly into *S. cerevisiae* EBY100. Western analysis demonstrated the presence of the recombinant protein too (Figure 2).

Three Ni-binding peptides were induced on the EBY100 cell wall surface by galactose induction. As shown in Table 2, the WT yeast was itself a unique bioadsorbent with a functional chelating ability for Ni(II), revealed by an observed sorption of 1.625 ± 0.004 g g^−1^ (dry weight) Ni(II). Concerning the molecular mechanism of nickel uptake in *S. cerevisiae*, it was suggested that nickel are transported into the vacuole by a proton-gradient-driven system. It was also shown that nickel resistance may share common mechanisms with iron and cobalt resistance, general stress response, and oxidative damage (Kucukgoze et al., 2013). The display of all Ni-binding peptides on the yeast cell wall enhanced the sorption of Ni(II) and produced valuable bioadsorbents. The combined expression of recombinant peptides by EBY100/pYD1-N123 resulted in the highest sorption of Ni(II), 2.602 ± 0.041 g g^−1^ (dry weight). Among the strains displaying a single peptide construct, the EBY100/pYD1-N1 showed the highest Ni(II) sorption of 2.235 ± 0.005 g g^−1^ (dry weight), a 37.51% increase compared to the WT strain. The sorption of Ni(II) by *S. cerevisiae* EBY100/pYD1-N2 cells [2.107 ± 0.003 g g^−1^ (dry weight)] was higher than that observed for EBY100/pYD1-N3 cells [1.912 ± 0.003 g g^−1^ (dry weight)].

**Table 2:** Sorption capability of different recombinants of *S. cerevisiae*.

Strains	(g g^−1^, dry weight)	Increased absorbing capability compared to EBY100/pYD1 (%)	Sorption of immobilization (g g^−1^, dry weight)	Increased absorbing capability compared to EBY100/pYD1 after immobilization (%)	Inhibition zone size (mm)
EBY100/pYD1-N123	2.603 ± 0.004	60.15	1.521 ± 0.007	79.40	1.21
EBY100/pYD1-N1	2.235 ± 0.005	37.51	1.205 ± 0.004	42.12	2.58
EBY100/pYD1-N2	2.107 ± 0.003	29.66	1.13 ± 0.004	33.22	3.05
EBY100/pYD1-N3	1.912 ± 0.003	17.62	1.042 ± 0.016	22.86	3.31
EBY100/ pYD1	1.625 ± 0.004		0.847 ± 7.451		4.52

A comparison of the strains expressing the Ni-binding peptides indicated that higher expression of Ni(II)-binding peptides corresponded to a greater number of Ni(II) bound. It is conceivable that surface-engineered yeast is able to tolerate more Ni(II) ions than the wild type.

### Immobilization

Because heavy metals are recalcitrant and do not disintegrate, their immobilization is an ideal remediation strategy. Immobilized microbial adsorbents can chelate metal ions on the cell surface, which is convenient for the recycling or sequestration of nickel ions, improving the efficacy of heavy metal waste treatment.

To determine the optimal yeast immobilization conditions and maximize the sorption of Ni(II) by yeast cells, based on the data obtained from the single factor study, the effects of three independent variables, including the concentrations of calcium chloride (1.5–2.5%) and sodium alginate (2.5–3.5%), as well as temperature (15–25°C) were investigated using an orthogonal test with three levels to optimize the immobilization of surface-engineered yeast (Zhang et al., 2017).

As shown in Table 3, the concentration of sodium alginate had the most positive effect on yeast immobilization, followed by the concentration of calcium chloride, which had a slightly important affect; temperature had the least affect. By using a range analysis, the optimal immobilization conditions for yeast were determined as 2.0% calcium chloride, 3.0% sodium alginate, and a temperature of 25°C. Under these conditions, the binding capability was 68.62%.

**Table 3:** Immobilization optimization of different recombinants.

No.	Calcium chloride (%)	Sodium alginate (%)	Temperature (°C)	Binding capability (%)
1	1.5	2.5	15	63.12
2	1.5	3	20	66.34
3	1.5	3.5	25	62.29
4	2	2.5	25	64.32
5	2	3	15	68.62
6	2	3.5	20	63.52
7	2.5	2.5	20	62.33
8	2.5	3	25	63.45
9	2.5	3.5	15	59.52
K1	63.667	63.000	63.000	
K2	65.000	65.667	63.000	
K3	61.333	61.333	64.000	
R	3.667	4.334	1.000	

**Figure 3:**
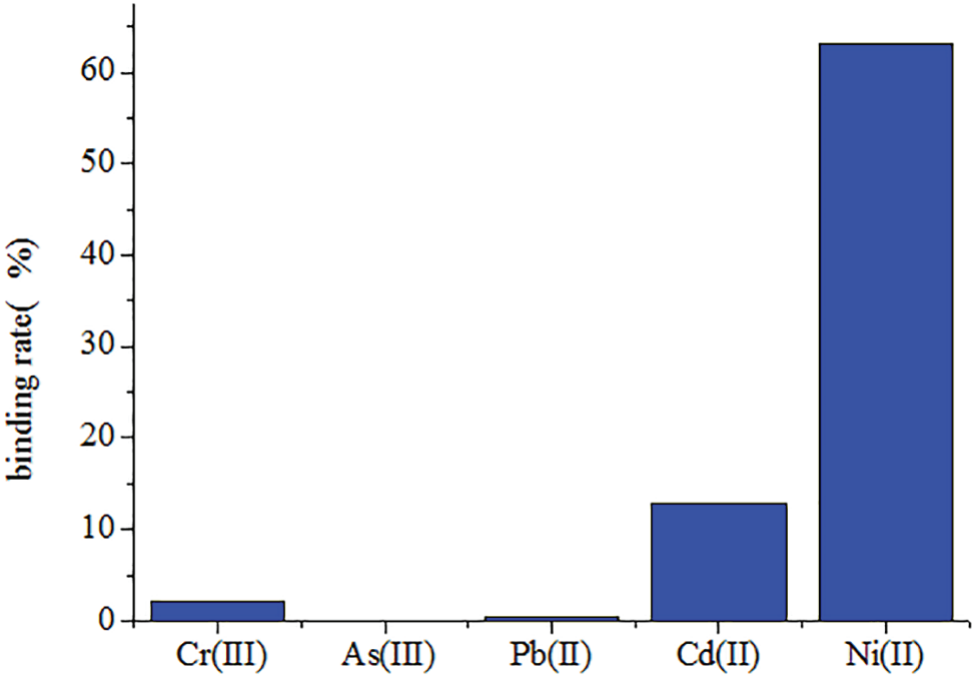
The to different heavy-metal ions selectivityof yeast recombinants.

The Ni(II)-binding ability among recombinant yeast was compared after immobilization (Table 2). The EBY100/pYD1-N123 strain exhibited the greatest Ni(II) sorption enhancement (79.40%) compared to the EBY100/pYD1 strain, with a maximum binding of 1.521 ± 0.007 g g^−1^ (dry weight) of Ni(II). The EBY100/pYD1-N1 strain adsorbed 1.205 ± 0.004 g g^−1^ (dry weight) Ni(II), an increase of 42.12% compared to the WT strain. The lowest Ni(II) sorption was observed for the EBY100/pYD1-N3 strain. One half binding capability of all strains after immobilization was decreased approximately compared with the same non-immobilized strains. The disc diffusion assay demonstrated that the largest inhibition zone size was occurring at control EBY100/pYD1, which is the least tolerance to Ni(II), and EBY100/pYD1-N123 has the most resistant to nickel ions. To verify the binding capability of Ni(II)-binding peptide, many heavy-metal ions were selected to test its selectivity. Figure 3 showed that Ni(II)-binding peptide showed no response to As(III) and a little sorption to Cr(III), and Pb(II), 12.75% sorption capability to Cd(II), verifying the high specificity for Ni(II).

## Conclusion

The display of Ni-binding peptides on yeast creates high-capacity bioadsorbents that are useful for the remediation of Ni-containing wastewater. Sorption through the cell surface is more powerful than intracellular sorption: (1) sorption through the cell surface enhances the binding of metal compared to the WT strain; (2) sorption through the cell surface makes it easier to recover metal ions from wastewater and does not require bacterial cell lysis; (3) sorption through the cell surface is feasible in even dead cells, since non-viable cells can adsorb heavy-metal ions by metabolism-independent surface binding rather than energy-dependent intracellular uptake.

## Author Contributions

HL was responsible for the Ni(II) sorption test. WD was responsible for the western blot analysis. GW was responsible for the cell immobilization. All authors contributed to revising the manuscript.

### Conflict of Interest Statement

The authors declare that the research was conducted in the absence of any commercial or financial relationships that could be construed as a potential conflict of interest.
